# Improved Sensitivity to Cerebral White Matter Abnormalities in Alzheimer’s Disease with Spherical Deconvolution Based Tractography

**DOI:** 10.1371/journal.pone.0044074

**Published:** 2012-08-31

**Authors:** Yael D. Reijmer, Alexander Leemans, Sophie M. Heringa, Ilse Wielaard, Ben Jeurissen, Huiberdina L. Koek, Geert Jan Biessels

**Affiliations:** 1 Department of Neurology, University Medical Center Utrecht, Utrecht, The Netherlands; 2 Image Sciences Institute, University Medical Center Utrecht, Utrecht, The Netherlands; 3 Vision Lab, Department of Physics, University of Antwerp, Wilrijk, Antwerp, Belgium; 4 Geriatric Department, University Medical Center Utrecht, Utrecht, The Netherlands; University of Maryland, College Park, United States of America

## Abstract

Diffusion tensor imaging (DTI) based fiber tractography (FT) is the most popular approach for investigating white matter tracts in vivo, despite its inability to reconstruct fiber pathways in regions with “crossing fibers.” Recently, constrained spherical deconvolution (CSD) has been developed to mitigate the adverse effects of “crossing fibers” on DTI based FT. Notwithstanding the methodological benefit, the clinical relevance of CSD based FT for the assessment of white matter abnormalities remains unclear. In this work, we evaluated the applicability of a hybrid framework, in which CSD based FT is combined with conventional DTI metrics to assess white matter abnormalities in 25 patients with early Alzheimer’s disease. Both CSD and DTI based FT were used to reconstruct two white matter tracts: one with regions of “crossing fibers,” i.e., the superior longitudinal fasciculus (SLF) and one which contains only one fiber orientation, i.e. the midsagittal section of the corpus callosum (CC). The DTI metrics, fractional anisotropy (FA) and mean diffusivity (MD), obtained from these tracts were related to memory function. Our results show that in the tract with “crossing fibers” the relation between FA/MD and memory was stronger with CSD than with DTI based FT. By contrast, in the fiber bundle where one fiber population predominates, the relation between FA/MD and memory was comparable between both tractography methods. Importantly, these associations were most pronounced after adjustment for the planar diffusion coefficient, a measure reflecting the degree of fiber organization complexity. These findings indicate that compared to conventionally applied DTI based FT, CSD based FT combined with DTI metrics can increase the sensitivity to detect functionally significant white matter abnormalities in tracts with complex white matter architecture.

## Introduction

Diffusion tensor imaging (DTI) based fiber tractography (FT) is currently the most widely used method to reconstruct fiber pathways in the brain, despite its well known limitations in regions with complex white matter architecture [1]–[3]. The common second-rank diffusion tensor model, however, is based on the assumption of Gaussian diffusion, which may not be valid in white matter voxels that contain so-called “crossing fibers” [4], i.e. complex fiber bundle architecture within a single voxel including two or more crossing, interdigitating or “kissing” fiber populations, or one fiber population with a bending or splaying architecture.

In the past decade, several advanced approaches for characterizing the intra-voxel diffusion profile have been developed to overcome the limitations of the second-rank diffusion tensor model [5]–[11]. One of these techniques, constrained spherical deconvolution (CSD) [9], is especially promising as it can offer a reliable reconstruction of multiple fiber orientation distributions within clinically feasible MR acquisition settings [8]. Notwithstanding the promising outlook, the CSD model has not yet been applied quantitatively to clinical populations due to the lack of robust diffusion metrics that can describe the underlying microstructure unambiguously.

We hypothesize that if CSD based FT is more accurate in reconstructing fiber bundle trajectories in regions with “crossing fibers”, it should be more sensitive to microstructural abnormalities underlying cognitive dysfunction than DTI based FT in these tracts. In tracts without “crossing fiber” regions, both methods should perform equally. To test this hypothesis we used a hybrid framework, in which CSD based FT is combined with conventional DTI metrics to assess white matter abnormalities in patients with early Alzheimer’s disease (AD). This allowed us to examine the microstructural properties of specific white matter pathways in relation to memory performance, while overcoming the well-known limitations of DTI based FT in regions with “crossing fibers”. We evaluated this CSD-DTI framework for two white matter tracts: one specifically selected because it contains many regions of “crossing fibers”, i.e. the superior longitudinal fasciculus (SLF) and one with only one fiber orientation, i.e. the midsagittal section of the corpus callosum (CC). Diffusion measures in these tracts have been previously shown to be altered in patients with AD compared to controls using tract based analyses [12] and to the AD-associated impairments in memory function [13].

In this paper, we examined whether CSD based FT combined with DTI metrics can increase the sensitivity to detect functionally significant white matter abnormalities in tracts with complex white matter architecture compared to conventionally applied DTI based FT.

## Methods

### 1. Ethic Statement

The medical ethics committee for research in humans of the University Medical Center Utrecht, the Netherlands has approved this research. Informed written consent was obtained from all participants. All clinical investigation has been conducted according to the principles expressed in the Declaration of Helsinki. Exclusion criteria were a history of stroke in the last 2 years, a history of stroke with subsequent cognitive deterioration, schizophrenia or other psychotic disorders, major depression, alcohol abuse, brain tumor, epilepsy or encephalitis. Incapacitated patients with a severe stage of AD, indicated by a clinical dementia rating score >1 [14] or a MMSE score <20 [15] were also excluded. The clinical dementia rating score was determined by the patients’ doctor and the diagnosis (Alzheimer/a-MCI) was established at a multidisciplinary meeting.

### 2. Participants

Twenty five patients (mean age 80.0±5.0 years, 48% male), 19 with early stage AD and 6 with amnestic mild cognitive impairment (a-MCI) were recruited via a memory clinic at the University Medical Center Utrecht. Probable or possible AD was diagnosed according to the National Institute of Neurological and Communicative Disorders and Stroke and the Alzheimer Disease and Related Disorders Association (NINCDS-ADRDA) criteria [16]. A-MCI was diagnosed according to the Petersen criteria [17]. Exclusion criteria were a history of stroke in the last 2 years, a history of stroke with subsequent cognitive deterioration, schizophrenia or other psychotic disorders, major depression, alcohol abuse, brain tumor, epilepsy or encephalitis. Incapacitated patients with a severe stage of AD, indicated by a clinical dementia rating score >1 [14] or a MMSE score <20 [15], were also excluded.

### 3. Data acquisition

MRI data were collected using a Philips 3.0 Tesla scanner (Intera, Philips, Best, the Netherlands). Diffusion MRI data were obtained using a single-shot spin echo EPI sequence with the following parameters: field of view = 220×220×120 mm^3^, 2.5 mm slice thickness (without gap), 48 slices, repetition time 6638 ms, echo time 73 ms, flip angle 90 degree, acquisition matrix 88×88 (in plane resolution of 2.5 mm) and reconstructed at 128×128, 45 isotropically distributed diffusion-sensitizing gradients with a b-value of 1200 s/mm^2^, and one b = 0 s/mm^2^ image [18]. The acquisition time was 5.32 min. Signal-to-noise ratio (SNR) within all WM voxels (FA>0.2) of the b = 0 s/mm^2^ image was on average 33.5 with a standard deviation of 11.7 [19].

### 4. Image processing

The DTI data sets were corrected for eddy current induced geometric distortions and subject motion by realigning the diffusion-weighted images (DWIs) to the b = 0 s/mm^2^ image with *Elastix* [20]. In this procedure, the diffusion gradients were adjusted with the proper b-matrix rotation as described by Leemans and Jones [21]. The diffusion tensor model was fitted using the RESTORE approach [22]. The DTI scans were transformed rigidly to MNI space in the motion–distortion correction procedure by using a single interpolation step (concatenation of transformation matrices) to maximize the uniformity of brain angulation across subjects [23].

### 5. Tractography

Standard deterministic streamline DTI [24] and standard CSD [25] based tractography were performed with the *ExploreDTI* software package (www.exploredti.com). We reconstructed the SLF and the CC using both FT methods with a uniform seed point resolution of 2 mm^3^ and a maximum deflection angle of 30 degrees. For the DTI based FT an FA threshold of 0.2 was applied. Analogously, the applied termination threshold for CSD based FT was a fiber orientation distribution (FOD) value of 0.1 (the harmonic degree of the estimated FOD coefficients was limited to 6) [8]. For this study we selected fiber tracts that were previously shown to be affected in MCI and AD [12], [13], [26] and either have a complex fiber architecture with crossing fibers or a single fiber population without crossing fibers. The SLF contains a relatively large number of voxels with multiple fiber orientations due to the crossing of the corona radiata and/or laterally projecting fibers of the CC and is therefore particularly susceptible to tracking errors caused by the second-rank diffusion tensor model [25]. By contrast, the midsagittal section of the CC contains mainly voxels with one fiber population and is expected to be less vulnerable to tracking errors.

**Figure 1:**
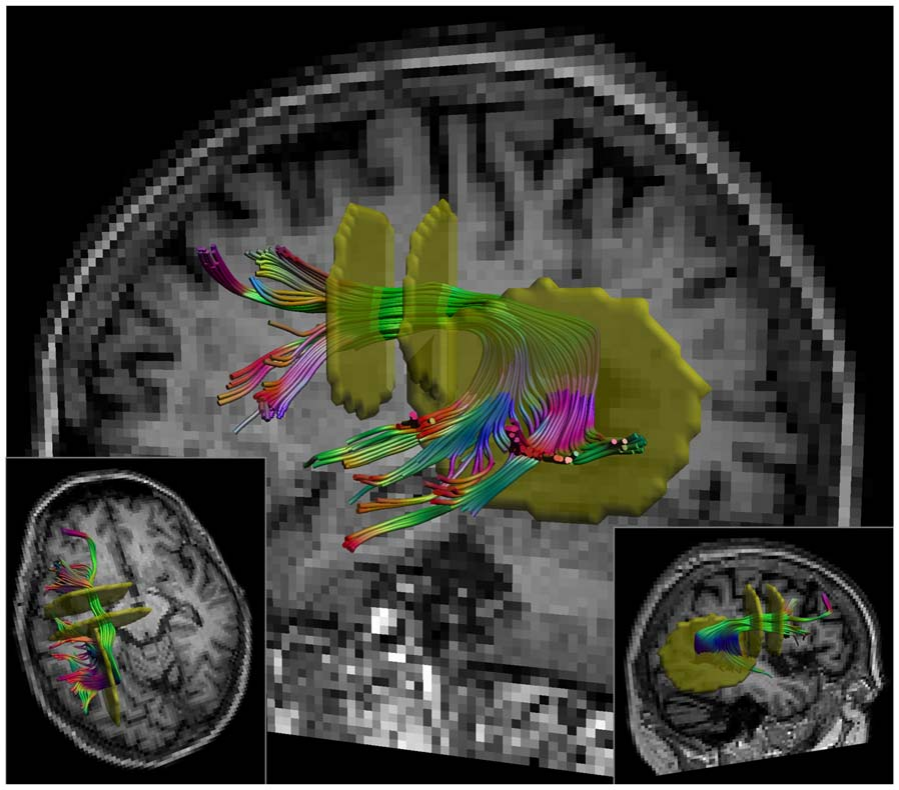
Selection of the superior longitudinal fasciculus (SLF). The SLF was selected using a multiple region of interest (ROI) approach. Two “AND” ROIs (shown in yellow) were placed on a coronal slice and one on a sagittal slice. Reconstruction was based on a standardized atlas of white matter tracts [28].

The SLF, including SLF II, III and the arcuate fasciculus [27], was reconstructed from the left hemisphere (all participants were right handed) based on a standardized atlas of white matter tracts [28]. For reconstruction of the SLF, a multiple region of interest (ROI) selection approach was used. In total, three “AND” ROIs were placed, two on a coronal and one on a sagittal slice (see Figure 1). In this ROI protocol, previously defined anatomical landmarks for slice selection and ROI placement were used to reduce subjectivity in fiber tracking [28]. High intra- and inter-rater reliability of manually segmenting fiber bundles has been demonstrated in previous studies (e.g. [29]–[31]).

**Figure 2:**
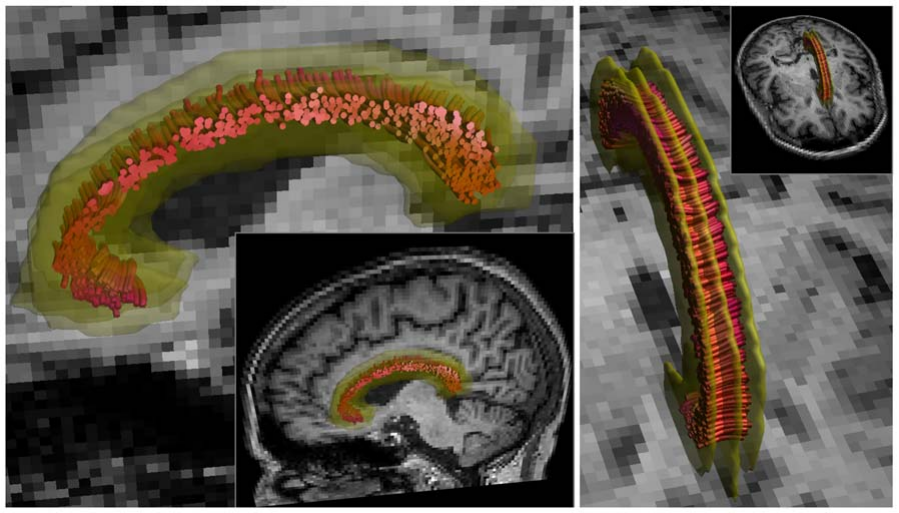
Selection of the medial segment of the corpus callosum (CC). The CC was selected using a multiple region of interest (ROI) approach. The median ROI was placed on the midsaggital plane in MNI space, and the two segment-selecting ROIs were drawn two voxels (4 mm) to either side of the midsagittal plane.

The CC was reconstructed as described previously [32]. In summary, only the midsagittal segment of the CC was selected to exclude regions of “crossing fibers” from the more laterally projecting pathways of the CC that intersect the corticospinal fiber trajectories (Figure 2). Note that as all data were analyzed in MNI space, the midsagittal slice could be determined reliably in all subjects.

**Figure 3:**
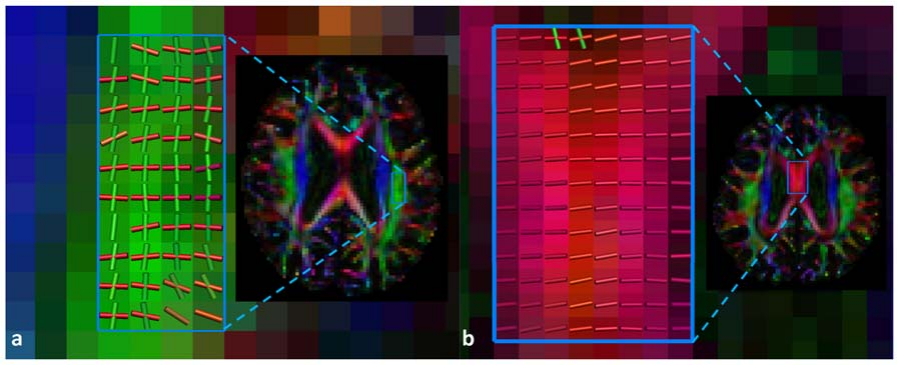
Fiber orientation distribution profiles estimated with the CSD method. a) two crossing fiber populations in voxels in the superior longitudinal fasciculus. b) one fiber population in the corpus callosum.

**Figure 4:**
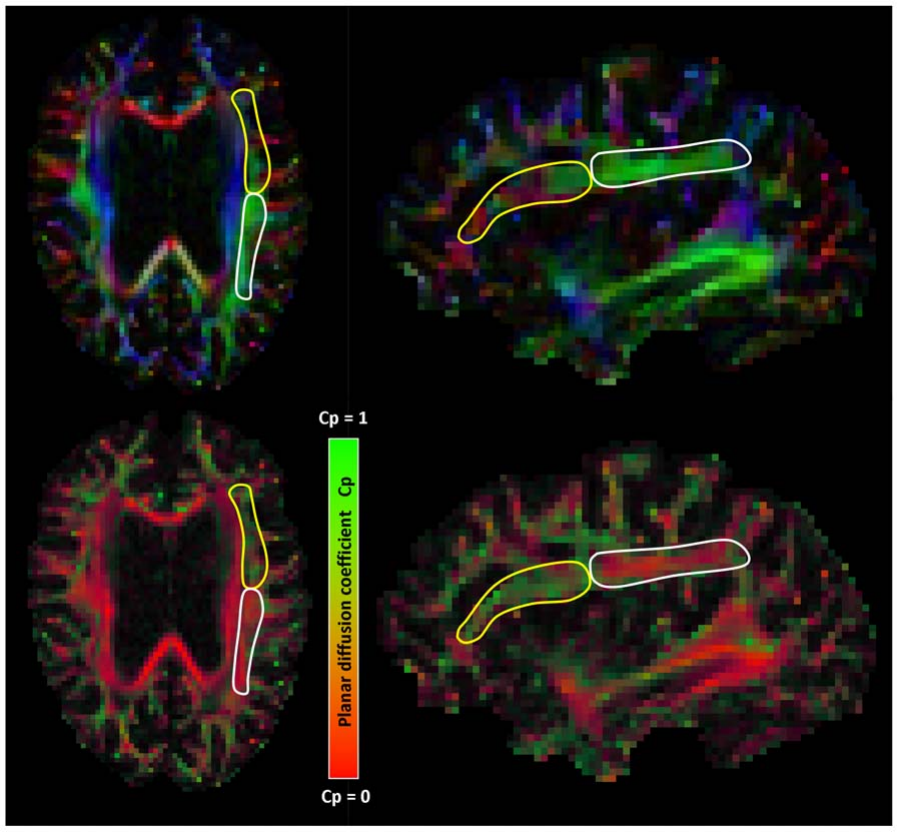
Crossing fiber regions in the superior longitudinal fasciculus (SLF). Sub-regions of the SLF marked on a directionally encoded color map (top row) and planar diffusion coefficient encoded (Cp) map (bottom row). The planar diffusion coefficient ranges from zero to one and is relatively high in voxels were the tensor has a disc-like shape, which is typically the case when two fiber populations “cross”. The white line marks a sub- region of the SLF containing voxels with relatively few “crossing fibers”, which is reflected by a Cp close to zero. By contrast, the more anterior sub-region of the SLF, marked in yellow, contains relatively many voxels with “crossing fibers”, due to crossing with the cortico-spinal tract and/or laterally projecting fibers of the corpus callosum. This is reflected by a Cp closer to one.

**Figure 5:**
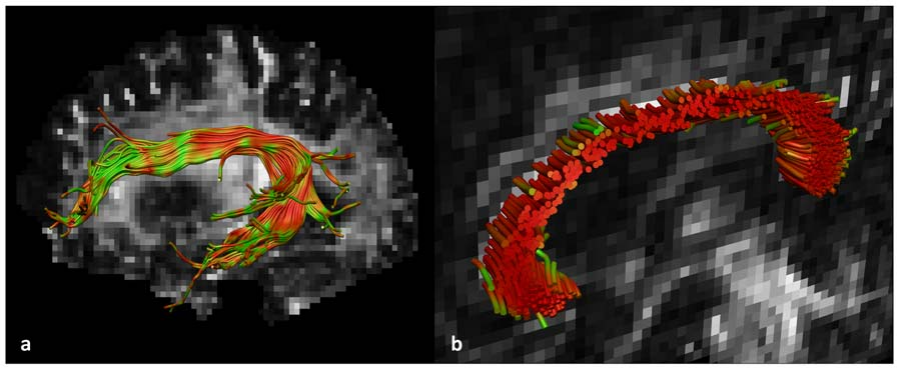
Crossing fiber regions reflected by the planar diffusion coefficient. The superior longitudinal fasciculus (SLF) and medial segment of the corpus callosum (CC) color coded according to the value of the planar diffusion coefficient (Cp) (for interpretation of the color coding see also fig. 3). The figure shows regions with “crossing fibers” reflected by a Cp close to one (green) and regions with relatively few “crossing fibers” reflected by a Cp close to zero (red). a) the SLF shows many regions with “crossing fibers” due to crossing with the cortico-spinal tract and/or laterally projecting fibers of the corpus callosum (CC) in frontal regions, and with the inferior longitudinal fasciculus in temporal regions. b) In the midsagittal segment of the CC one fiber population predominates.

Diffusion parameters: fractional anisotropy (FA), mean diffusivity (MD), radial diffusivity (DR), axial diffusivity (DA), and the normalized planar diffusion coefficient (λ_2_−λ_3_/λ_1_) [33] were obtained for each tract. The planar diffusion coefficient was used to quantify the degree of fiber complexity in regions with “crossing fibers” [33], [34]. The planar diffusion coefficient ranges from zero to one and is relatively high in voxels where the tensor has a disc-like shape (i.e. the first and second eigenvalue are almost equal and larger than the third eigenvalue). This is typically the case when two fiber populations “cross” or “kiss” [34]–[37] (Figures 3, 4, and 5). It is important to note, that although the planar diffusion coefficient and FA or MD are both an index of the tensor shape, they are not directly related: the FA can be similar in voxels with linear or planar diffusion [38]. By contrast, the planar diffusion coefficient provides a geometric description of the tensor and hence is more specific to the fiber configuration [34].

### 6. Cognitive testing

All patients underwent a standardized cognitive assessment including a test assessing verbal memory: the Raven’s Auditory Verbal Learning Task (RAVLT) [39]. Because deficits in learning and memory are the main cognitive symptoms of (early) AD, we selected memory performance as the primary functional measure of disease severity. Immediate and delayed recall scores of the RAVLT were transformed into z-scores and averaged to obtain one composite memory score.

### 7. Statistical analyses

Differences in configurational tract characteristics (volume, length) after DTI and CSD based FT were analyzed with paired-samples T-test. The composite memory score and diffusion measures were all normally distributed. The relation between mean DTI metrics (FA, MD, DA and DR) and memory performance was evaluated using linear regression analysis adjusted for age, sex, and level of education. Differences in the relation between DTI metrics and memory obtained with DTI- versus CSD-based FT was calculated using Steiger’s Z-statistic for dependent correlations [40].

Because crossing fibers affect the tensor estimation [35], [41], [42], the relation between DTI metrics and cognition cannot be reliably assessed in regions with “crossing fibers”. We therefore adjusted for the degree of “crossing fibers” in a second model, by including the planar diffusion coefficient of the diffusion tensor model [33], reflecting the degree of fiber complexity, as a covariate. As such, we limited the adverse effect of “crossing fibers” on the relation between DTI metrics (i.e. FA, MD, DR, DA) and cognition. This modulating effect is expected to be most pronounced in combination with CSD based FT in tracts with crossing fibers, since more voxels with “crossing fibers” will be included using this method.

To examine the possibility that the relation between diffusion measures and memory performance is affected by tract volume [43], we ran a separate model with age, sex, education level and estimated tract volume as covariates.

## Results

### 1. CSD vs. DTI Based FT

**Figure 6:**
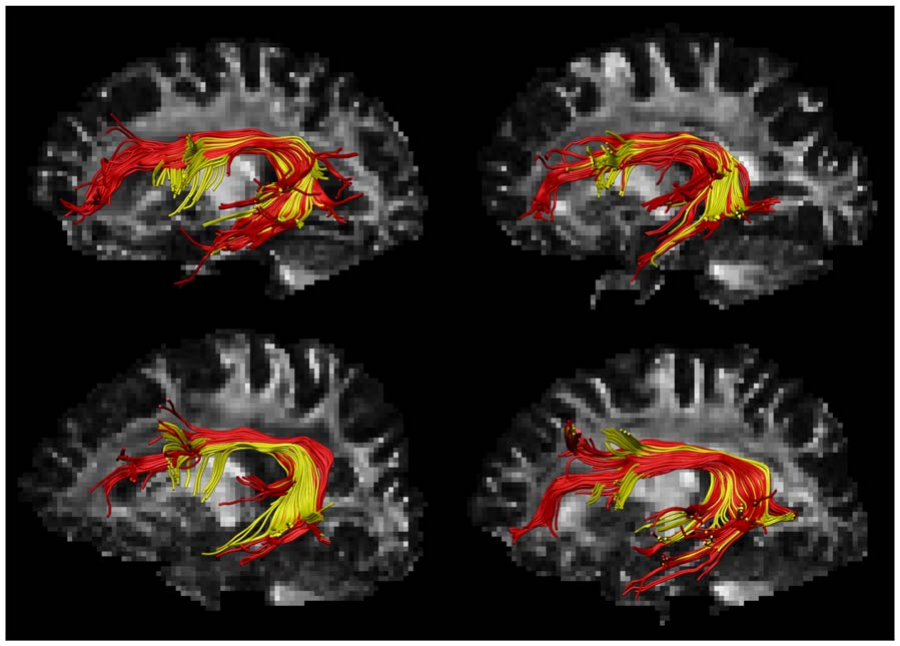
Segmentation of the superior longitudinal fasciculus (SLF) with DTI and CSD based fiber tractography. Segmentation of the SLF in four patients reconstructed with DTI (yellow) and CSD (red) based fiber tractography (FT). Delineation of the SLF resulted in larger and longer pathways with CSD compared to DTI based FT. With the DTI method, fibers of the SLF were more likely to terminate at crossings between the SLF and the cortico-spinal tract in frontal regions and between the SLF and the inferior longitudinal fasciculus in temporal regions.

Figure 6 shows the SLF of four representative patients reconstructed with DTI and CSD based FT. In all patients, the tract volume of the SLF was larger with CSD than DTI based FT (mean tract volume ± SD (cm^3^) CSD: 19.88±5.25; DTI: 10.10±2.78; p<0.001). In 75% of the patients the tract length was longer with CSD compared to DTI based FT (mean tract length ± SD (mm) CSD: 109.2±10.9; DTI: 98.3±13.6; p<0.001). The approximate tract volume of the CC segment was also larger for all patients with CSD compared to DTI based FT (mean tract volume ± SD (cm^3^) CSD: 9.59±0.98; DTI: 7.03±0.94; p<0.001).

### 2. Association between DTI Metrics of the SLF and Memory Performance with CSD and DTI Based FT

For the SLF, lower FA values of the SLF were associated with worse memory performance for both FT methods, but this association was only statistically significant for CSD based FT (standardized regression coefficient (95% CI) DTI: 0.39 (0.01; 0.78); p = 0.054, CSD: 0.41 (0.02; 0.81); p = 0.042) (Table 1, model 1). MD was not significantly associated with cognitive performance. Additional adjustment for the planar diffusion coefficient, reflecting the degree of fiber organization complexity, did not change the results for the DTI based method (Table 1, model 2). By contrast, the relation between the FA of the SLF and memory performance in combination with CSD based FT became stronger after adjustment for the planar diffusion coefficient (0.53 (0.14; 0.92); p = 0.010). The modulating effect was even more pronounced for the MD: the regression coefficient became three times as large after adjustment of the planar diffusion coefficient (−0.55 (−1.07; −.02); p = 0.044). Post hoc analyses showed that memory performance was related with DR but not with DA measures (DR: −.55 (−.0; −.11); p = 0.018, DA: −0.22 (−0.92; 0.48); p = 0.511). Adjustment for tract volume did not change these relations significantly (data not shown). Importantly, the relation between DTI parameters and memory was significantly stronger for CSD- compared to DTI based FT, for MD (Z = 4.38; p<0.0001), DR (Z = 4.18; p<0.0001), and DA (Z = 2.02; p = 0.02), but not FA (Z = 1.55; p = 0.06) (Tabel I, model 2). Correlation plots of the adjusted and unadjusted data are presented in Figure S1 and S2 respectively.

**Table 1:** Association diffusion parameters of the SLF and memory performance.

	DTI based tractography		CSD based tractography	
	Beta (95% CI)	p-value	Beta (95% CI)	p-value
*Model 1*				
FA	0.39 (0.01; 0.78)	0.054	0.41 (0.02; 0.81)	0.042
MD	−0.18 (−0.62; 0.25)	0.383	−0.16 (−0.58; 0.27)	0.461
Axial diffusivity	0.01 (−0.45; 0.47)	0.967	0.08 (−0.39; 0.53)	0.737
Radial diffusivity	−0.27 (−0.68; 0.15)	0.195	−0.26 (−0.67; 0.15)	0.205
*Model 2*				
FA	0.36 (−0.04; 0.76)	0.074	0.53 (0.14; 0.92)	0.010
MD	−0.23 (−0.66; 0.20)	0.283	−0.55 (−1.07; −0.02)^a^	0.044
Axial diffusivity	−0.10 (−0.59; 0.40)	0.690	−0.22 (−0.92; 0.48)^a^	0.511
Radial diffusivity	−0.27 (−0.68; 0.14)	0.178	−0.55 (−1.0; −0.11)^a^	0.018

Data are presented as standardized regression coefficients with 95% CI.

Model 1: adjusted for age, sex, level of education.

Model 2: Model 1+ adjustment for the planar diffusion coefficient, reflecting the degree of fiber organization complexity.

a Regression coefficient is significantly larger for CSD compared to DTI based tractography, assessed with Steiger’s Z-statistic.

### 3. Association between DTI Metrics of the CC and Memory Performance with CSD and DTI Based FT

We also assessed the relation between diffusion parameters and cognitive performance in a tract without “crossing fibers”: the midsagittal segment of the CC. The FA of the CC was not significantly associated with memory performance with either tractography method, whereas a trend was observed for an association between memory and mean MD (DTI:−0.40 (−0.80; 0.002); p = 0.051, CSD: −0.37 (−0.78; 0.04); p = 0.074) (Table 2, model 1). After adjustment of the planar diffusion coefficient, the relation between the FA, MD and memory performance became stronger. However, the regression coefficients remained comparable between both tractography methods (all p<0.05; Table 2, model 2). Post hoc analyses showed that memory performance was related with DR and not with DA measures, with comparable regression coefficients with DTI and CSD based FT (−0.51 and −0.50 respectively). Again, adjustment for tract volume did not change the results significantly (data not shown).

**Table 2:** Tabel 2. Association diffusion parameters of the CC and memory performance.

	DTI based tractography		CSD based tractography	
	Beta (95% CI)	p-value	Beta (95% CI)	p-value
*Model 1*				
FA	0.31 (−0.10; 0.72)	0.134	0.27 (−0.15; 0.68)	0.197
MD	−0.39 (−0.80; 0.002)	0.051	−0.37 (−0.78; 0.04)	0.074
Axial diffusivity	−0.34 (−0.75; 0.08)	0.104	−0.35 (−0.76; 0.07)	0.097
Radial diffusivity	−0.38 (−0.79; 0.02)	0.061	−0.34 (−0.76; 0.07)	0.099
*Model 2*				
FA	0.54 (0.06; 1.02)	0.031	0.50 (0.01; 1.00)	0.045
MD	−0.45 (−0.86; −0.04)	0.035	−0.45 (−0.88; −0.02)	0.040
Axial diffusivity	−0.33 (−0.76; 0.10)	0.122	−0.34 (−0.77; 0.08)	0.109
Radial diffusivity	−0.51 (−0.94; −0.08)	0.022	−0.50 (−0.96; −0.05)	0.031

Data are presented as standardized regression coefficients with 95% CI.

Model 1: adjusted for age, sex, level of education.

Model 2: Model 1+adjustment for the planar diffusion coefficient, reflecting the degree of fiber organization complexity.

Regression coefficients obtained with DTI and CSD based FT did not differ significantly, assessed with Steiger’s Z-statistic.

## Discussion

This is the first report on the application of CSD based FT to detect white matter abnormalities in patients with (early) AD. Our results indicate that 1) CSD based FT in combination with DTI metrics significantly increased the sensitivity to detect a relation between white matter abnormalities and memory performance in a tract with “crossing fibers” (SLF); and 2) the relation between DTI metrics and memory was comparable between both FT methods in a tract without “crossing fibers” (midsagittal section of the CC).

In line with our expectations, fibers of the SLF were more likely to terminate in regions with “crossing fibers” with DTI-based FT. By contrast, with CSD based FT the SLF continued beyond these crossings to more temporal and dorsal frontal regions, which is in line with descriptions from autopsy studies [27], [44] and with previous papers using spherical deconvolution based FT [45]. Our results extend these findings by showing that improvement of fiber tract segmentation increases the sensitivity to white matter abnormalities within the tract.

The adverse effects of “crossing fibers” on the interpretation of diffusion measures such as MD and FA have been previously demonstrated (e.g. [35], [41], [42]), but their impact on the detection of white matter abnormalities is not known. A number of studies have found contra-intuitive results in regions with “crossing fibers”, such as the centrum semiovale, demonstrating increased FA values in patients compared to controls [46], [47] and a negative correlation between FA and cognitive function [48], [49]. These unexpected findings may result from degeneration of one pathway, with relatively sparing of the crossing pathway. For example in AD, late-myelinating white matter tracts such as the SLF have been shown to degenerate at an earlier stage than tracts that myelinate early in life [50], [51]. This is supported by results from a recent study showing an *increased* mode of anisotropy in patients with MCI compared to controls only in areas where the SLF intersects the projection pathways [46].

Voxels with “crossing fibers” are more likely to be included with CSD based FT. We therefore used the planar diffusion coefficient as a covariate to overcome the confounding effects of these “crossing fibers” on the diffusion metrics in relation to cognition. If two fiber populations within a voxel “cross” or “kiss”, the shape of the diffusion tensor becomes more planar (disc-like). As a result, a voxel with intact crossing fibers can have a similar FA value compared to a voxel with a degenerating non-crossing fiber population. However, the planar diffusion coefficient between these voxels will be different [38]. Our results showed that co-varying for the planar diffusion coefficient effectively increased the strength of the relation between DTI metrics in the SLF and memory performance. As expected, this modulation was most pronounced in combination with CSD based FT. Adjusting for the planar diffusion coefficient also increased the association between DTI metrics and memory in the CC, despite the lack of any interdigitating fiber pathways. Possibly, this finding can be explained by the presence of residual partial volume effects between the dorsal part of the CC and the adjacent cingulum bundles. Partial volume effects also affect the tensor estimation and the measures derived from it [34], [35] and may therefore confound the relation between DTI metrics and cognition in the same way.

The effects of DTI metrics on cognitive performance were more prominent for DR than for DA, suggesting that the observed association is more likely driven by myelodegeneration than by a loss of axonal integrity [52]. However, it should be noted that many more cellular characteristics, such as hydration, cell packing density and fiber diameter could cause the observed changes in diffusion measures [53]–[55] and that the interpretation of these diffusivity measures can be far from trivial [41].

Our study has a number of limitations. One is the modest sample size, which may have decreased our sensitivity to detect a relation between structure and function. Still, we were able to replicate previously observed associations between diffusion measures and AD severity [56], [57]. Second, FT based segmentation is laborious and time consuming. However, the advantage over automated voxel based or atlas based analyses is that it is less sensitive to individual anatomical differences, imperfect registration, and smoothing errors [58]–[60]. Moreover, averaging of the diffusion metrics along a fiber bundle reduces the variance in diffusion measures and thereby increases the power to detect more subtle WM changes. On the other hand, very localized changes along a fiber bundle, for instance, only in the structure’s anterior part, may not be picked up when the anterior and posterior parts are combined. To limit the number of comparisons we focused in the present study on two major tracts, but future studies should demonstrate whether these findings extend to other fiber pathways containing complex and simple white matter architecture known to be affected in AD [61] or other neurological diseases.

Finally, the use of the planar diffusion coefficient as a quantitative measure to characterize “crossing fibers” may be valid in cases were two fiber bundles intersect or overlap, but may not be directly applicable in regions where three or more fiber bundles intersect. Although previous work reported that no more than two fiber populations could be observed in the SLF [62], there is still no consensus on the prevalence of multiple fiber populations [4]. In this context, future studies are needed to investigate this issue in detail and more specific measures for “crossing fibers” need to be developed to improve the sensitivity for detecting white matter abnormalities in clinical populations and to make the interpretation of structure-function relationships less ambiguous.

### Conclusion

Since DTI based FT fails in regions with “crossing fibers”, more accurate methods to characterize the microstructural properties of fiber pathways are in need. Here we showed that CSD based FT combined with standard DTI metrics increases the sensitivity to detect functionally significant white matter abnormalities in a tract with “crossing fibers” in patients with early AD compared to DTI based FT. The use of a hybrid CSD-DTI framework is therefore a promising tool to detect functionally significant white matter changes in regions with complex white matter architecture.

## Supporting Information

Figure S1 **Adjusted correlations between diffusion parameters of the SLF and memory performance.** Top row: the relation between FA/MD and memory with DTI based fiber tractography. Bottom row: the relation between FA/MD and memory with CSD based fiber tractography. FA/MD and memory are expressed as standardized residuals after adjusting for age, sex, level of education, and the planar diffusion coefficient (Table 1, model 2).
(TIF)

Figure S2 **Unadjusted correlations between diffusion parameters and memory performance.** Top row: the raw FA values of the SLF (left) and medial segment of the CC (right) with DTI based fiber tractography. Bottom row: the FA of the SLF (left) and medial segment of the CC (right) with CSD based fiber tractography.
(TIF)
